# Multimodal Assemblies for Prefacing a Dispreferred Response: A Cross-Linguistic Analysis

**DOI:** 10.3389/fpsyg.2021.689275

**Published:** 2021-09-27

**Authors:** Simona Pekarek Doehler, Hilla Polak-Yitzhaki, Xiaoting Li, Ioana Maria Stoenica, Martin Havlík, Leelo Keevallik

**Affiliations:** ^1^Center for Applied Linguistics, Institute of Language Sciences, University of Neuchâtel, Neuchâtel, Switzerland; ^2^Department of Hebrew Language, University of Haifa, Haifa, Israel; ^3^Department of East Asian Studies, University of Alberta, Edmonton, AB, Canada; ^4^Czech Language Institute, Czech Academy of Sciences, Prague, Czechia; ^5^Division of Language, Culture and Interaction, Department of Culture and Society, Linköping University, Linköping, Sweden

**Keywords:** preference organization, gaze, epistemic markers, conversation analysis, turn-prefacing, multimodality

## Abstract

In this paper we examine how participants’ multimodal conduct maps onto one of the basic organizational principles of social interaction: preference organization – and how it does so in a similar manner across five different languages (Czech, French, Hebrew, Mandarin, and Romanian). Based on interactional data from these languages, we identify a recurrent multimodal practice that respondents deploy in turn-initial position in dispreferred responses to various first actions, such as information requests, assessments, proposals, and informing. The practice involves the verbal delivery of a turn-initial expression corresponding to English ‘I don’t know’ and its variants (‘dunno’) coupled with gaze aversion from the prior speaker. We show that through this ‘multimodal assembly’ respondents preface a dispreferred response within various sequence types, and we demonstrate the cross-linguistic robustness of this practice: Through the focal multimodal assembly, respondents retrospectively mark the prior action as problematic and prospectively alert co-participants to incipient resistance to the constraints set out or to the stance conveyed by that action. By evidencing how grammar and body interface in related ways across a diverse set of languages, the findings open a window onto cross-linguistic, cross-modal, and cross-cultural consistencies in human interactional conduct.

## Introduction

Participants in face-to-face social interaction deploy multiple resources for building actions in mutually accountable ways. A pioneer in the field, Charles Goodwin (e.g., Goodwin, 1981, 2017), has demonstrated that human social interaction is a complex ecology of vocal and bodily visual resources that people draw on to build joint actions. While some multimodal resources are used in concert in *ad hoc* manners to accomplish local interactional tasks, there are also more or less routinized configurations of language and the body (“multimodal packages,” Hayashi, 2005; Kärkkäinen and Thompson, 2018, or “gestalts,” Mondada, 2014) – that is, recurrent constellations of vocal and bodily conduct that are routinely deployed to accomplish particular interactional tasks. In line with Goodwin’s (2013, p. 12) observations of how participants to social interaction dynamically “assemble” such multiple resources to create actions, we refer to such configurations as “multimodal assemblies.” We are interested in exploring the *methodic* character of such assemblies, i.e., recurrent body-language constellations that ‘go together’ and are put to use in systematic ways as part of interactants’ accountable practices for conducting social interaction (see also Keevallik, 2013; Li, 2014; Pekarek Doehler, 2019; Skogmyr Marian, 2021, forthc), and we seek to uncover how these compare cross-linguistically. It is our belief that a better documentation of the commonalities of multimodal behavior of humans around the globe may bring us closer to understanding the possibly shared organizational principles of social interaction in particular, and of human social conduct in general.

In order to develop such an understanding, research has started to explore the ways speakers shape their conversational actions cross-linguistically and cross-culturally (cf. Stivers et al., 2009; Dingemanse and Floyd, 2014; Floyd et al., 2020; see below). In this paper, we focus on speakers of five geographically and culturally distinct languages, from five different language (sub)families: Czech (Slavic branch of the Indo-European language family), French and Romanian (Romance branch of the Indo-European language family), Hebrew (Semitic branch of the Afro-Asiatic language family), Mandarin (Sino-Tibetan language family).^1^ We analyze how speakers of these languages employ equivalents of the construction ‘I don’t know’ in combination with precise bodily conduct, namely gaze averted from the recipient, in a precise sequential environment, namely turn-initial position in responsive actions. We document that this multimodal assembly serves as a preface to dispreferred responses, such as refusing a proposal or not answering an information request. And we shed light on the sequential location of respondents’ averting their gaze – relative to the prior speaker’s turn and to the response itself – and then possibly returning it to the prior speaker in the course of the response (which we refer to as ‘gaze trajectory’).

The Estonian audio Excerpt (1) provides an initial sense of the *linguistic* phenomenon at hand, i.e., how ‘I don’t know’ type of expressions can work to launch a dispreferred response. In this excerpt, Ene responds to Anni’s question-word question as to the whereabouts of a crib.

**Figure:**
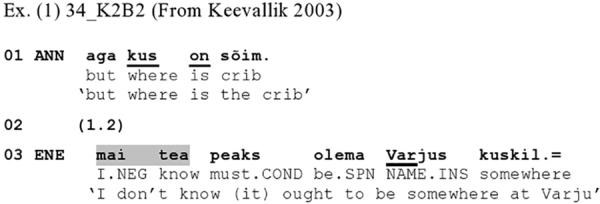


The response comes in late – a typical trait of dispreference (cf. Pomerantz, 1984; Sacks, 1987) – and is prefaced by a morphophonologically reduced *mai tea* ‘I dunno’ (the full form would be *ma ei tea*), which is furthermore prosodically latched to the following phrase; it hence stands as a preface to what follows rather than a turn-constructional unit (TCU) in itself. In what follows, Ene offers a strongly non-committal response of the type that Stivers (2010) has treated as a non-answer response, and hence a dispreferred response.^2^ While the *mai tea* may here also work as a pre-positioned hedge, downgrading the speaker’s commitment to her response (see Weatherall, 2011, on English), in this sequential environment it prominently projects that the incipient response departs from the relevancies issued by the sequence-initiating question: It alerts the recipient to the upcoming of a dispreferred next action.

A related working is shown in the French Excerpt (2), where *ch’pas* ‘dunno’ projects a dispreferred response (note that here it cannot be heard as an epistemic hedge), conjointly with the preceding *be:n* ‘well.’ In this excerpt, Marie asks Julie why she would rather choose to learn languages by means of immersion than standard instruction (l.01):

**Figure:**
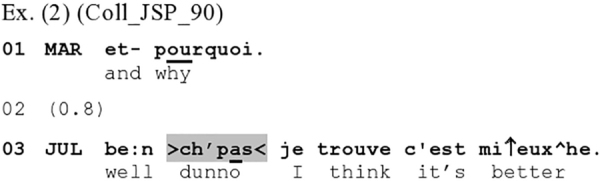


Julie’s response (l.03) bears typical traits of a dispreferred response (Sacks, 1987), i.e., one that does not align with the terms of the sequence-initial action: It starts with a delay, and is *be:n* ‘well’-prefaced (cf. Davidson, 1984; for French ‘ben’ see Bruxelles and Traverso, 2001; Persson, 2020). This is followed by a morphophonologically reduced and prosodically backgrounded (speedup of tempo) variant of *je sais pas* ‘I don’t know,’ namely *ch’pas* ‘dunno,’ which is prosodically packaged together with what follows into a single prosodic unit, rather than standing as an independent unit. These features concur to display the *ch’pas* as a preface to the subsequent response *je trouve c’est mieux* ‘I think it’s better’ (the turn-final *he* is a non-lexical vocalization, produced through strong exhalation). Importantly, this response does not conform to the terms of the question, as Julie simply re-affirms that she prefers immersion (l.03): She offers a non-answer response, and hence a dispreferred response (Clayman, 2002; Stivers and Robinson, 2006). In this sequential environment, the > *ch’pas* <, here conjointly with the *be:n* ‘well,’ alerts the recipient to the dispreferred nature of the upcoming next action.

While the above excerpts illustrate the verbal side of the interactional practice that we investigate in this contribution, we show in the remainder of this paper that speakers deploy such verbal conduct recurrently as part of a multimodal assembly. The assembly comprises a morphosyntactic construction corresponding to English ‘I don’t know’ and its variants (henceforth: IDK) that is prosodically produced as part of the emerging TCU and is coupled with the speaker’s gaze averted from recipient. The respondent’s gaze itself follows an on-line trajectory, comprising aversion most often either within the transition space between turns or coinciding with response onset (and the production of IDK), and then return to the prior speaker in the course of the response. Sequentially, the grammar-body-assembly is recurrently found in turn-initial position in dispreferred responsive actions. Based on multimodal conversation analysis, we provide cross-linguistic evidence showing that [IDK + gaze aversion] serves as a routinized multimodal resource for prefacing a dispreferred response: It indexes incipient resistance to the constraints set out or to the stance conveyed by the coparticipant’s prior action. While our findings converge with earlier studies that documented an association of gaze aversion with dispreference in the specific context of responses to polar questions in English (Kendrick and Holler, 2017; Robinson, 2020; see section “Background” below), they also amplify these by showing that the focal practice (a) pertains to a wide range of sequence types, i.e., is not limited to responses to polar questions, and (b) holds across a diverse set of languages.

In what follows, we first present the background (see section “Background”) and the data used for this study (see section “Data and methods”), and then turn to multimodal analysis of selected data excerpts (see section “Analysis”) documenting the occurrence of the focal multimodal assembly as a preface to dispreferred responses to questions (see section “Prefacing dispreferred responses to questions”) and to a range of other types of sequence-initial actions (see section “Prefacing dispreferred responses to proposals, assessments, and informings”). We end with discussing our findings and drawing conclusions regarding the interactional logic of the practice as well as how grammar and body interface across languages (see section “Discussion and conclusion”).

## Background

Preference organization is a basic organizational feature of social interaction – a “formal apparatus” (Sacks, 1987) pertaining to the sequential concatenation of actions. Basically, there are sequence initial actions that call for responses of two opposed types among which one tends to be favored over the other. So-called ‘dispreferred’ next actions are those that are “uncooperative, disaffiliative or otherwise discordant” (Clayman, 2002, p. 230), such as refusing an offer or not answering a question. So-called ‘preferred’ next actions are those that are most cooperative, allow the interaction to move forward and align with the action which they respond to Sidnell (2011, p. 81), such as granting a request or answering a question.^3^ The preference status of actions is reflected in their design, most centrally in their timing and more generally in what Sacks (1987) referred to as their ‘contiguity’: Preferred next actions tend to be delivered right away, while dispreferred actions tend to be delayed and pushed further back in the turn by means of pre-beginnings (such as *uhm* or inbreaths), hedging, and turn-prefaces (Sacks, 1987), including turn-initial particles such as *well* in English (Davidson, 1984; Heritage, 2015), *ben* in French (Bruxelles and Traverso, 2001; Persson, 2020) or *nu/nå* across a range of European languages (Auer and Maschler, 2016). That is, participants deploy a set of conventionalized practices through which they accomplish alternative types of responsive actions. Turn-initial position is particularly relevant to the preference status of responsive actions, as it is in this position that the dispreferred nature of these may be projected (e.g., Davidson, 1984; Pomerantz, 1984; Deppermann, 2013; Heritage, 2015; Heritage and Sorjonen, 2018).

In addition to turn-initial elements, the timing between the end of an initiating action and the beginning of its responsive action has been documented to project the preference status of responsive actions. Measuring the silence between the initiating and the responsive turns at talk in invitation, offer, request, suggestion, and proposal sequences in a corpus of English telephone conversations, Kendrick and Torreira (2015) show that dispreferred responses are delayed significantly longer than preferred responses (median = 561 ms vs. 269 ms; Kendrick and Holler’s, 2017 findings suggest that these times are shorter for responses to polar interrogatives). Bögels et al. (2015, using experimental methods) and Bögels et al. (2020, studying telephone conversations) further demonstrate that recipients change their expectations regarding the valence of a response based on the duration of silence between initiating actions and responsive actions: Recipients expect preferred responses after a short gap and dispreferred responses after a longer gap (roughly after 300 ms). Furthermore, there is initial evidence for cross-linguistic convergences in this patterning of preferred and dispreferred response-timing and the related expectations. Roberts et al. (2011) used experimental methods to show that in American English (see also Roberts et al., 2006), Italian, and Japanese, listeners perceive a response that comes in late as an indicator of a disagreement with an assessment or of an unwillingness to comply with a request.

In a nutshell, then, as Pomerantz and Heritage (2013, p. 228) put it: “The turn shapes through which initiating and responding actions are enacted represent interpretive resources” that index the preference status of an action. However, how multimodal conduct is relevant to preference organization has remained largely unexplored.

There is some evidence that disaligning or disaffiliative^4^ responsive actions in general are associated with respondent’s gaze aversion. Kidwell (2006) reports that recipient’s gaze-withdrawal after a directive may be seen as an act of resistance, and Haddington (2006) finds evidence that respondent’s gaze withdrawal from a prior speaker is associated with divergent stance. This is in line with Kendon’s (1967, p. 48) earlier observation that mutual gaze between speaker and recipient appears to decrease in non-affiliative and non-cooperative interactions. There are, to our knowledge, only two investigations directly addressing gaze in dispreferred responsive actions, both focusing on responses to polar questions in English. Based on quantitative and qualitative analysis of conversations recorded in a soundproof room, and using eye-tracking methodology, Kendrick and Holler (2017) found that preferred responses (defined as those responses that conform to the preference set up by the grammatical format of the yes-no question), tend to be produced with gaze toward the recipient (61%), while dispreferred responses are most frequently produced with gaze averted from the recipient (82%); in the latter case recipients’ gaze aversion tends to start after the first possible completion point of the question. Robinson (2020) presents a quantitative and a qualitative study of responses to information-seeking polar questions that confirms the association of gaze aversion with dispreference: Respondents producing dispreferred responses shift gaze away from questioners in 73% of the cases at some point between the beginning of the question and the beginning of the response (while they do so only in 21% of the preferred responses).

In light of the above findings on gaze, three issues deserve to be explored in more detail. One key open question is (i) whether the documented association of gaze aversion and dispreference can be found with dispreferred responses in contexts other than polar questions. Also (ii) the on-line trajectory of gaze conduct in dispreferred responses deserves attention, namely in terms of the sequential start of aversion (during the sequence-initial turn, in the transition space between turns, or coinciding with the response onset) and of possible return of gaze to recipient. And (iii) it remains to be investigated how far findings based on (American) English data may be valid for other languages and cultures.

While existing work has evidenced that gaze plays a crucial role in organizing and regulating interaction (Goodwin, 1981), for instance in regard to turn-taking (Kendon, 1967) or sequence organization (Rossano, 2012), cross-cultural consistency or variation in gaze conduct has so far gained only limited attention in research on social interaction. And this is so despite important intensification of comparative conversation analytic research across situations (e.g., Heritage and Clayman, 2010), cultures (Sidnell, 2009; Floyd et al., 2020), and time (Pekarek Doehler and Deppermann, 2021). The existing cross-linguistic/cultural studies of gaze in interaction have mainly focused on question-answer adjacency pairs. In their comparative analysis of question-answer sequences in conversational data from 10 languages, Stivers et al. (2009) found that questioner’s gaze affects the timing of responses: Responses were delivered earlier if the questioner was looking at the recipient while the question was asked. Rossano et al. (2009) investigated gaze in question-answer sequences in conversational data in Italian (from northern Italy), Yélî Dyne (Papua New Guinea), and Tenejapan Tzeltal (Mexico). They found that questioners look at recipients more often than vice-versa, but that recipients’ gaze conduct is less consistent cross-culturally. One of their results directly relates to our focal issue, namely that « lack of […] recipient gaze [on questioner] is a good predictor of lack of response after questions » (p. 220). This again begs the question as to whether recipient’s gaze averted from prior speaker is a more general feature of dispreference, i.e., if it can be found across a variety of sequence types.

As to the linguistic construction investigated in this paper, IDK has been shown to be associated with disaffiliative or dispreferred actions in two ways. For one thing, in its literal uses as a claim of no knowledge, IDK has been considered as a non-answer response, i.e., a dispreferred response in itself, that offers an account for not providing an answer (Stivers and Robinson, 2006). As such, it can be a means for indicating a problem with the question (Keevallik, 2011), resisting a line of questioning (Hutchby, 2002; Keevallik, 2011), resisting offers, proposals and invitations (Keevallik, 2011), avoiding an overt disagreement (Tsui, 1991, pp. 612–617), or deferring a dispreferred response (Helmer et al., 2016). For another thing, and different from the fully epistemic uses, there are also particle- (or: marker-)like uses of IDK that have been suggested to be associated with dispreference. These typically (see ex. 1 and 2 above) show formal reduction (e.g., English *dunno*) as well as semantic bleaching (concretely: loss of epistemicity) as typical features of routinization (for English: Bybee and Scheibmann, 1999; Scheibman, 2000). Schegloff (1988, p. 445; see also Schegloff, 1996) observed that *I dunno* can be one of a range of delay components in a dispreferred response. In a similar vein, Keevallik (2003, 2011) observed that Estonian *mai tea* ‘I don’t know’ (and its variant *ei tea*) may be used in disaligning actions giving dispreferred answers in an indirect manner, as well as signaling the speaker’s uncertainty toward the produced content. Maschler (2012, 2017) found that Hebrew (‘*ani) lo yode*‘*a/yoda*‘*at* ‘(I) don’t know.M/F’ (mainly its reduced form *loyde*‘*a/loydat*) may function as discourse markers changing the course of talk. Lindström and Karlsson (2016) point out that Swedish *jag vet inte* ‘I don’t know’ in doctor-patient interactions is a pragmatic marker that frames responsive turns as resisting the interlocutor’s question. Pekarek Doehler (2016) found that in French turn-initial *chais pas* ‘dunno’ projects a dispreferred response, whereas in mid-turn position it tends to function as an epistemic hedge and in turn-final position it serves as a turn- or sequence-closing device.

The above work provides evidence for turn-initial uses of IDK in responsive turns in Estonian, Hebrew, Swedish and French, for projecting a disaligning or dispreferred response. A systematic analysis of the uses of IDK in other languages, however, is still lacking. Also, we know little about the bodily visual behaviors possibly concurrent with IDK. Pekarek Doehler (2019) documents how participants’ embodied conduct, and specifically gaze, systematically differs between two distinct particle-like uses of French *chais pas* ‘dunno’ in turn-final position in sequence-initial actions: IDK plus gaze aversion serve as a practice to withdraw one’s own prior action, while IDK plus gaze on recipient, in the very same sequential position, is a practice to invite recipient’s response. To our knowledge this is the only existing study scrutinizing how IDK interfaces with bodily conduct in social interaction. In addition to the three issues raised under (i) through (iii) above, the question remains as to (iv) how the linguistic construction IDK combines with gaze as part of a practice for prefacing dispreferred responses. The present study documents the recurrence and robustness of this practice, and shows that it is generalizable across a variety of sequential contexts and languages.

## Data and Methods

The data for this study consist of video-recorded interactions between two to five participants. Most stem from conversations between friends/colleagues and family. The Czech data comes from TV talk shows but converges with the data in the other languages in that it consists of unscripted interactions that unfold spontaneously. As the latter data comes from the public domain, it neither needs anonymization nor consent for publication.^5^ Names in all other data have been anonymized, and informed consent has been obtained from all participants for use and publication of the materials, including the video frames. Cross-linguistic comparability across the datasets is based on our focus on a precise sequential environment: IDKs in turn-initial position in responsive actions.

We do not have space here for a structural description of the languages studied (but see Dryer and Haspelmath, 2013), and therefore limit ourselves to a succinct presentation of the IDK construction in these languages. Table 1 shows the conventional full and the reduced forms of IDKs across the languages (in alphabetic order)^6^.

**TABLE 1:** Forms of IDKs in the data in five languages.

Language	‘Canonical’ form	Reduced form
Czech	**(já) nevím** I NEG-know.1SG.PRS canonical form **[nεi 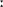 m]** with a long vowel i:, which is a phonological distinction in Czech	**[nεv 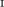 m]** - > change in vowel pronunciation and reduced length, often change in consonant pronunciation: [υ] instead of [v]
French	**je (ne) sais pas** I NEG know.1SG.PRS NEG	**j’sais pas chais pas ch’pais** - > 1 person pronoun and the start of ‘know’ are amalgamated to various degrees
Hebrew	**’ani lo yode’a/yoda’at** I NEG know.SG.PRS.M/F	**’ani lo yde’a ‘anloyde’a loyde’a lo yoda’at loyda’at loydat** - > various degrees of reduction, including elision of first person pronoun and amalgamation of the negator and ‘know’
Mandarin	**(wo) bu zhidao** I NEG know.1SG.PRS	**bu zhidao**
Romanian	**(eu) nu ştiu** I NEG know.1SG.PRS	**nu ştiu**

Table 2 sums up the extent of the data (hours of recording per language) and the IDK tokens found therein. For Romanian, due to unavailability of existing data transcribed according to CA standards, we have so far only a small corpus that has been specifically recorded and transcribed for the purpose of this study; the results for this language hence call for further confirmation based on a larger dataset.

**TABLE 2:** Overview of IDK tokens (without complement) occurring in the data from the five languages.

Language(and hours of recording)	(I) Total IDK (without a complement)	(II) Turn-initial *particle-like* IDK in responsive actions	(IIIa) Turn-initial particle-like IDK in *dispreferred* responsive actions	(IIIb) Thereof: tokens with respondent’s *gaze averted* from prior speaker
Czech (130 h)	776	47	**43**	**39**
French (20 h)	184	32	**21**	**19**
Hebrew (8 h)	108	29	**22**	**20**
Mandarin (17 h)	83	36	**31**	**28**
Romanian (1 h35)	31	4	**2**	**2**

*The focal tokens for this study appear in bold.*

We established an initial inventory, for each language, of all instances of IDK occurring without a complement (column I, Table 2). We then identified amongst these all instances containing a turn-initial *particle*- (marker-)like use of IDK in *responsive actions*, independently of a precise sequence type. That is, we discarded all IDKs that were full epistemic disclaimers (e.g., IDKs accomplishing a non-answer response through a claim of no-knowledge), and this was motivated by the fact that full epistemic disclaims can accomplish dispreferred actions in themselves and are hence functionally different from the particle-like uses analyzed here, which preface such actions but do not accomplish them (see section “Background” above). We included as ‘turn-initial’ tokens those occurrences that were either not preceded by any vocal tract sound or were preceded by pre-starts (e.g., inbreaths, clicks, *euh:* or *hum* and the like) or particles (e.g., *ben* ‘well’). We then conducted multimodal sequential analysis of the collection for each language and discarded all instances that were either not or not clearly categorizable as dispreferred responses (see Robinson, 2020 for continua of preference) or in which the respondent’s gaze conduct was not identifiable from the video captures. This left us with the tokens indicated in column IIIa, Table 2. Among these, we identified those instances that were coupled with respondent’s gaze averted from the prior speaker (column IIIb), which amounted to roughly 90% of the cases for each language (except: 100% of the two tokens for Romanian). The association of particle-like IDK plus gaze aversion occurring in turn-initial position in dispreferred responses was hence found to be a strong tendency across the languages, with only a few exceptions for each of these (4 for Czech, 2 for French, 2 for Hebrew, 3 for Mandarin, 0 for Romanian). The quantitative results regarding the recurrence of respondent’s gaze aversion in dispreferred responses converge with the tendencies observed by Kendrick and Holler (2017) and Robinson (2020) for the specific case of responses to polar questions in English (see above). We then organized, for each language, the collection in terms of sequence types; the association of gaze-aversion with dispreference was found across a range of sequence types that we discuss below (excerpt for Romanian for which we have only 2 target occurrences due to a limited dataset; see above).

In section “Analysis,” we present selective excerpts from our data to illustrate the convergent ways in which IDK and gaze tend to be coupled in projecting a dispreferred response. Data excerpts in Czech, French, and Romanian use CA conventions for transcription of verbal conduct (Jefferson, 1984); excerpts in Mandarin are transcribed using the GAT-2 transcription system (Selting et al., 2011); excerpts in Hebrew are transcribed according to the Santa Barbara transcription method (Du Bois, 2012). The multimodal transcription conventions follow Mondada (2018).

## Analysis

In this section we document speakers’ use of IDK in conjunction with gaze averted from the recipient in prefacing a dispreferred response. The following sequential pattern is observed, in which gaze aversion may coincide with the IDK or precede it:

A: sequence-initiating action
(gap)
B: *IDK and gaze aversion* + other turn elements that form a responsive action

The findings across the languages studied show that IDK in this use is semantically bleached (it does not work as a claim of no knowledge and is not treated as such) and tends to be morpho-phonologically reduced or otherwise prosodically downgraded (e.g., by speed-up of tempo or lower volume). These features suggest grammaticalization into a particle-like element (Bybee and Scheibmann, 1999 for English; Maschler, 2012, 2017 for Hebrew; Keevallik, 2003 for Estonian, Pekarek Doehler, 2016 for French). The IDK-prefaced turn is often delayed and/or sometimes prefaced by particles such as *no*/*ben* ‘well,’ sound objects such as *pff* or clicks as further typical traits of dispreferred responses. This provides cross-linguistic evidence for [IDK + gaze aversion] working as a composite multimodal resource prefacing a dispreferred response. We first discuss question-answer sequences (see section “Prefacing dispreferred responses to questions”) as a case of canonical adjacency pair, and then extend the analysis to other action sequences, namely those including proposals, assessments and informing (see section “Prefacing dispreferred responses to proposals, assessments, and informings”).

### Prefacing Dispreferred Responses to Questions

Question–answer sequences have attracted much attention in research. Questions can implement a range of actions (request for information or confirmation, repair, etc.; e.g., Stivers, 2010). Answers are preferred over non-answers (Clayman, 2002; Stivers and Robinson, 2006); with polar questions, confirming answers are preferred over disconfirming answers (Sacks, 1992), and type-conforming answers (i.e., structurally ‘fitted’ answers: yes/no) are preferred over non-conforming ones (Raymond, 2003). Overall, preferred responses are delivered faster than dispreferred responses (e.g., Raymond, 2003; Stivers et al., 2009; Stivers, 2010; Heritage, 2012). In the data, IDK-prefaced dispreferred responses are found in responses to both question-word and polar questions that work as requests for information.

#### Responses to Question-Word Questions Seeking Information

The Romanian Excerpt (3) provides a first illustration. Greta and Ana are sharing a break at the end of their working day. Reporting on an event that happened during the day, Ana mentions the fact that an employee was on vacation (l.03–04), upon which Greta asks ‘where’ (l.05). Up to line 6, participants maintain mutual gaze.

**Figure:**
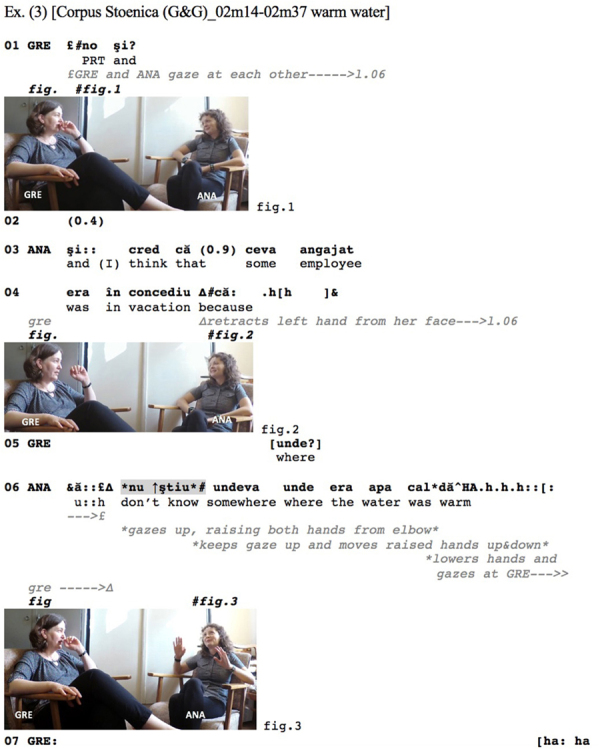


At line 06, Ana’s responsive turn starts with an initial hesitation marker and a subsequent *nu ştiu* ‘don’t know,’ both concurring to delay the response proper. This is followed by the type of strongly non-committal response discussed above that stands as a dispreferred response (cf. Stivers, 2010, p. 2778): ‘somewhere where the water was warm.’ While the IDK possibly also functions here as a pre-positioned hedge (Keevallik, 2011; Weatherall, 2011), downgrading the speaker’s commitment to her incipient response, it is centrally implicated in the sequential concatenation of turns and actions: it projects that the incipient response departs from the relevancies established by the sequence-initiating question. Noteworthy is the fact that the respondent shifts her gaze away from the co-participants, up into the air; the onset of the gaze shift coincides with the *nu ştiu* ‘don’t know,’ and gaze is maintained averted throughout the responsive action, turning back at Greta only at its very end (l.06). Interestingly, in the course of the response, the semiotic quality of the gaze appears to change: The gaze, coupled with Ana’s hands spread out raising up (Figure 2) and then down (l.06), heightens the non-committal nature of her response as it is combined with a shrug (see also her raised eyebrows, cf. Streeck, 2009, p. 189) – a pragmatic gesture (Kendon, 2004) conferring disengagement (Streeck, 2009, p. 189): It is as if Ana was enacting ‘don’t ask me’.^7^ The search ends on Ana laughing (l.06), which is met by Greta’s laughter (l.07).

A similar IDK-gaze pattern can be observed in the Hebrew data shown in Excerpt (4). Alex and Dotan, two friends at the beginning of their master’s degree in biology, are talking about Alex’s new position as a practice lecturer. Bracha, a friend of Dotan, sits behind the camera. After Dotan had inquired about how Alex’s office hour with the students went, Alex responds ‘it’s not such fun’ (l.01) upon which Dotan inquires ‘why’ (l.03):

**Figure:**
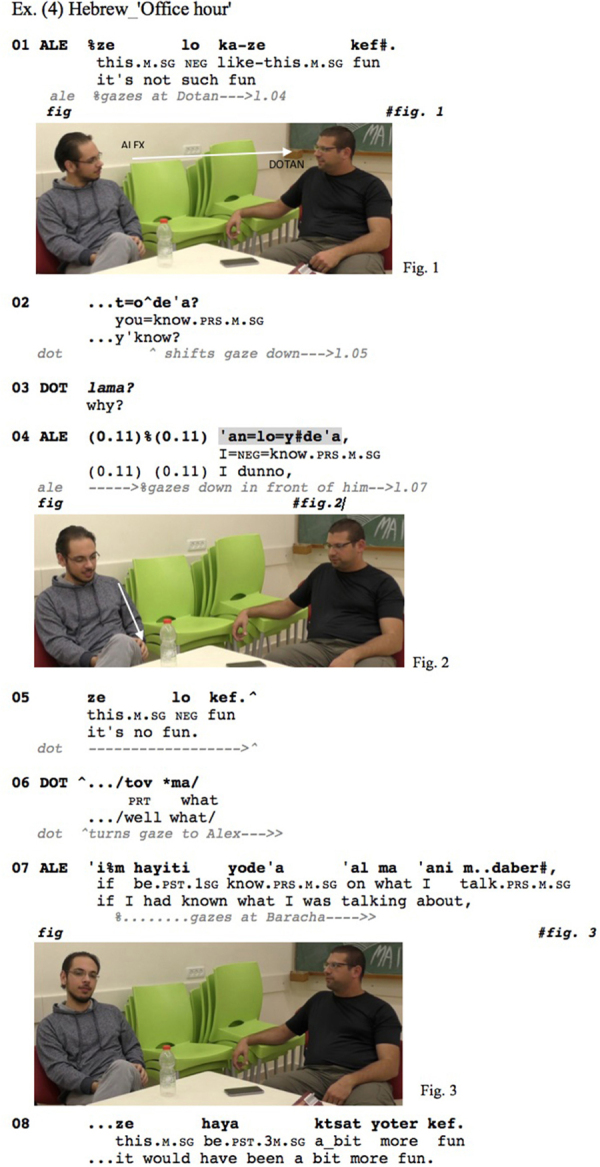


Just like in the preceding excerpt, Dotan’s ‘why’ (l.03) is a question-word question that accomplishes a request for information. Alex responds with a reduced (see Table 1 above) *anloyde*‘*a* ‘I dunno’-prefaced response. Even though the ‘*anloyde*‘*a* token occurs in a separate intonation unit (and is marked as such according to the Santa Barbara transcription conventions, Du Bois, 2012), there is no pause following it and it is produced with a continuing intonation contour signaling that there is more to come, thus the two intonation units ‘…I dunno, it’s no fun.’ (l.04–05) are delivered as a single TCU. Alex’s response clearly does not conform to the terms of the question: Instead of providing an account for why it’s not fun, Alex just repeats that it is no fun (l.05), which is an upgraded version of his prior negative assessment ‘it’s not such fun’ (l.01). He then elaborates in a non-serious way on the conditions under which it would have been ‘a bit more fun,’ namely if he had known what he was talking about (l.07). So, again we have a dispreferred response that is prefaced by a morphophonologically reduced IDK, which is part of the same TCU as the subsequent response. This is coupled with the respondent averting his gaze from the prior speaker, right in the transition space after the ‘why’ question (l.03–04), and maintaining it through the ‘*anloyde*‘*a* and the rest of the turn, only at the end of which he re-directs his gaze to the co-participant (l.07).

**FIGURE 1:**
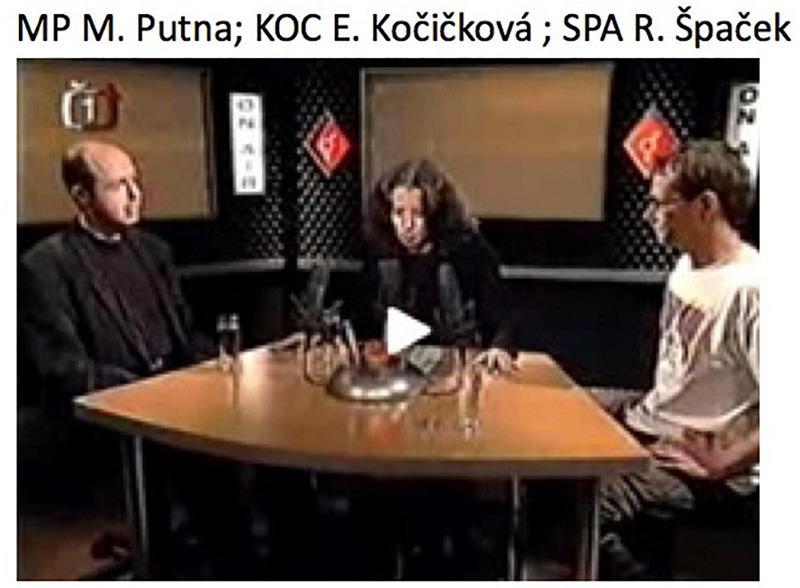
Spacial arrangement of participants in the TV show.

Similar features are observable in Excerpt (5), in French. Marie just mentioned that the condition for her to move abroad for a job would be that her boyfriend comes along. Pat then delivers a question-word interrogative, asking what if her boyfriend (*il* ‘he’) did not come along (l.01). After Marie starts a delayed and *bon* ‘well’-prefaced response (l.03–04), Pat adds that the job in question would represent Marie’s chance of a lifetime (l.05), thereby re-launching his inquiry in more dramatic terms: His *c’est la chance de ta vie* ‘it’s the chance of your life’ works to renew the relevance of his initial question, thereby increasing the pressure on the recipient to provide an answer.^8^ While Marie’s and Pat’s gazes meet during this re-launch (l.05, Figure 1), Marie averts her gaze already during the ensuing gap, slightly rising it to gaze over Pat’s right shoulder, then keeps it averted while delivering her IDK-prefaced response (l.07, Figure 2), and returns it to Pat only in the further course of her response (l.08):

**Figure:**
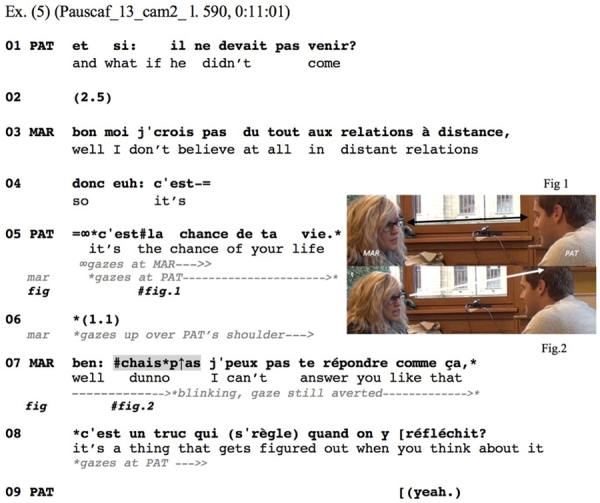


Marie’s response (l.07–08) comes in late and is *ben* ‘well’-prefaced (cf. Davidson, 1984; Heritage, 2015), both of which concur to indicating an upcoming dispreferred response. The IDK (here: *chais p→as)* itself shows strong morphophonological reduction (see Table 1 above), and slight rise of pitch projecting continuation. These features concur to displaying it as a preface to some incipient action, rather than as accomplishing an action in itself, and specifically as a preface to a dispreferred next action: Marie provides a non-answer response that resists the terms of the question by claiming inability to answer (l.07; note that in this context French *répondre* corresponds to English ‘answer’); thereby she explicitly claims her response to be a response, and treats the immediately preceding action, preferred in a declarative format (l.05), as pursuing an answer on her part to Pat’s prior question. This is followed by an account for her inability to offer an answer (l.08), which is ultimately accepted by Pat (l.09).

The way the turn is designed is quite conclusive. The turn-initial *ben:* ‘well’ and the subsequent *chais p→as* operate a division of labor. French ‘ben’ is a multifunctional particle, which, in turn initial position in second pair parts, has been found to accomplish such various things as introducing dispreferred responses, indexing contestation of the relevancy of a prior question, prefacing an incipient topical shift or opening a conclusive remark (Bruxelles and Traverso, 2001; Persson, 2020). It is hence an “elusive” (Heritage, 2015) particle similarly to English ‘well’ that, in responses to questions, can signal various types of departures, ranging from dispreference or non-straightforwardness, through resistance regarding the relevance of a question, to steering away from what precedes. In the present case, we have a response that clearly does not conform to the terms of the question. In this context, the IDK more specifically than the *ben:* alerts the recipient to the dispreferred nature of the upcoming response and not only to its non-straightforwardness or to some other moving away from the expected next. The delayed turn-start, the *ben:*, the *chais p?as* prefacing, and the speaker’s gaze aversion hence work in concert to project the incipient response as departing from the agenda set up by the preceding request for information.

Excerpts 3 through 5 illustrate a recurrent [IDK + gaze aversion] pattern projecting a dispreferred response in a precise sequential location and action context, namely in responses to seeking information: 3 and 4 in response to a question-word question, in 5 in response to a declarative format that re-does a prior question-word interrogative. The assembly of IDK plus gaze conduct shows a distinct on-line trajectory: gaze aversion either occurs prior to the delivery of IDK (in the transition space) or coincides with its start, but not later than IDK; the respondent’s gaze is then maintained averted from the prior speaker during IDK and into the responsive turn, and typically returns to the prior speaker toward the end of the response. As illustrated in the next sub-section, this temporal assembly of multiple resources and its interactional working is also found in responses to polar questions seeking information (and in other action environments, see section “Prefacing dispreferred responses to proposals, assessments, and informings”).

#### Responses to Polar Questions Seeking Information

Excerpt (6), from a Czech TV talk-show, illustrates the case of a dispreferred response to an information-seeking polar question, formatted as a declarative ending in a tag (l.01). Marek Eben interviews the Czech writer Ivan Klíma, who, when he was a child, had been imprisoned in a concentration camp for 4 years, together with his siblings and parents. When Klíma asks Eben whether he had also experienced torment in his life (l.01), Eben responds that his suffering was in no way comparable to Klíma’s (l.02).

**Figure:**
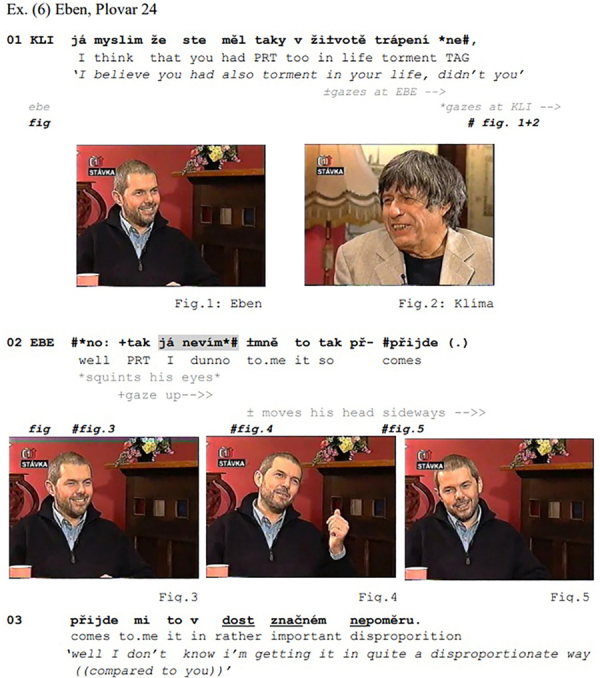


Through its declarative format, the turn-initial *já myslim* ‘I believe’ and the final tag *ne* ‘no,’ Klíma’s question (l.01) projects a confirming response as a preferred next (see Sacks, 1992; Heritage, 2010)^9^. Furthermore, the lexical element *trápení* ‘torment’ confers a sense of strong misery that the speaker suggests the recipient has suffered. Yet, the respondent’s response is neither type-conforming, nor does it do confirmation. Rather, the respondent downplays the importance of the misery he himself experienced, thereby discarding the presupposition encoded in the question, while, at the same time conferring sympathy with the profound torment Klíma must have gone through.

Just like in the excerpts cited above, the response bears typical traits of a dispreferred action: While it comes in without a gap, the generic turn-initial *no:* ‘well’ prefacing^10^ is here lengthened through sound-stretch, and is followed by the particle *tak*, both contributing to the delay of the actual response. This is enhanced by the subsequent *já nevím* ‘I dunno,’ which is reduced in form, as Eben pronounces it as [nε
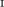
m] instead of [nεvi
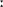
m]. The *nevím* is here preceded by the first person pronoun *já* ‘I’ – albeit Czech is a pro-drop language –, which works as a further delay component, in addition to the preceding *no tak ‘*well so,’ all of which concur with the formally reduced [nε
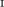
m] to project the incipient response as dispreferred. Just as in the preceding excerpts, the IDK is uttered with the respondent’s gaze averted from the questioner: During Klíma’s question, participants had established mutual gaze (l.01; Figures 1, 2).^11^ However, exactly at the onset of his reply, Eben stops looking at Klíma, squints his eyes (Figure 3), raises his head slightly and looks away – first up (Figure 4), then down (Figure 5). At the same time, he performs a pragmatic gesture by raising his left hand while further turning his gaze up (Figure 4) and, when doing so, proclaims *no tak já nevím* ‘well so I dunno.’ (The camera shifts from Eben to Klíma after *pøijde* in line 03, so that Eben’s further gaze conduct remains undocumented).

The Hebrew Excerpt (7) shows Eden and Lital, two friends, sitting together at a café, drinking cold coffee. Suddenly Eden suggests that they will have something to eat (l.01):

**Figure:**
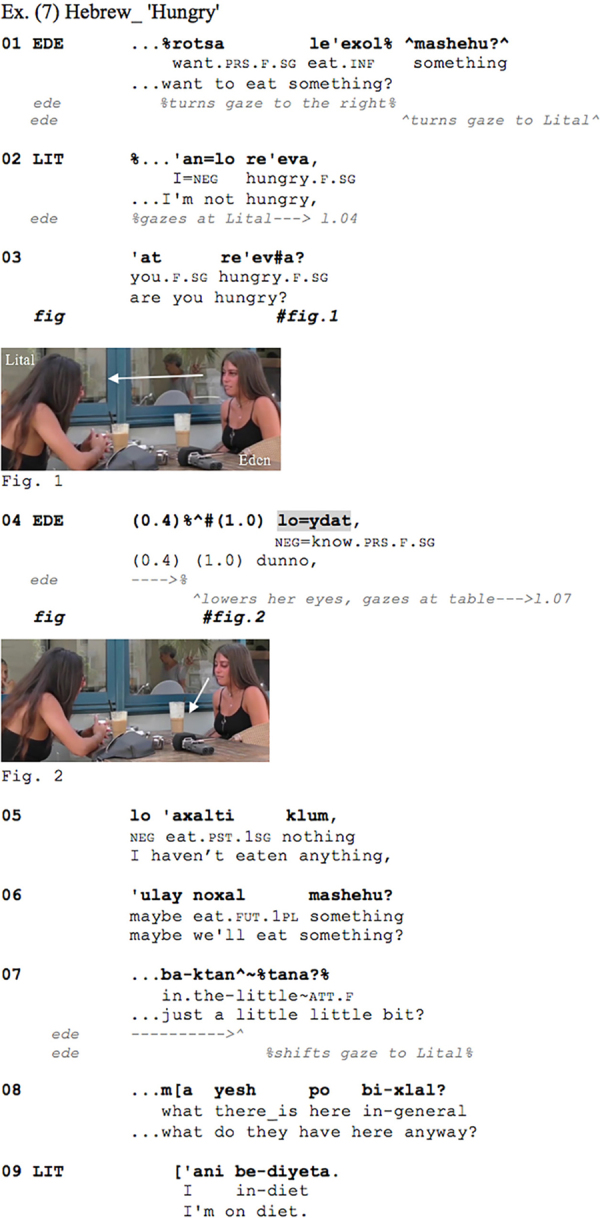


Eden’s initial question may be seen as a suggestion to order food, yet Lital responds straightforwardly declaring that she is not hungry (l.02), thereby conveying that she is not interested in ordering food. She then immediately adds a polar question asking Eden ‘are you hungry’ (l.0.3). Eden’s response to this question comes in with a strong delay during which she lowers her gaze, averting it from Lital, and subsequently produces the morphophonologically reduced token of *loydat* ‘dunno’(l.04) further delaying her response (cf. Schegloff, 1988, p. 445), while keeping her gaze averted and returning it to the questioner only way into her turn (l.07). Note that even though *loydat* is in a separate intonation unit (Du Bois, 2012 transcript), it is still delivered as a single TCU with the subsequent intonation unit. Here again the IDK plus gaze aversion occur as a preface to a dispreferred response. For one thing, Eden’s response is not type-conforming with regard to the yes-no question (Raymond, 2003). Furthermore, some kind of opposite stance between the participants is also in play, as Eden’s admitting that she has not eaten anything (l.05) and suggesting that they could order ‘just a little little bit’ contrast with Lital’s prior affirmation that she herself is not hungry, and by implication, does not ‘want to eat’ (l.01), i.e., order food. This is further foregrounded by the fact that Eden repeats her (implicit) suggestion from line 01, this time using first person plural denoting both her interlocutor and herself (l.06): ‘maybe we’ll eat something?’.

#### Intermediate Summary

The excerpts discussed in this section showed a recurrent verbal-embodied practice for projecting dispreferred responses to information- and confirmation-seeking questions: speakers use IDK in turn-initial position, typically in a morphophonologically reduced form, combined with gaze aversion, thereby foreshadowing the non-conformity of the upcoming response to the sequence-initiating question. In the data, these responsive turns show consistently dispreferred action-turn-shapes: delayed turn-starts, particles such as *ben, no* ‘well,’ lengthening, hesitation markers such as *u:h* and/or vocalizations such as *phhh* or clicks preceding the IDK; all these elements push the actual response further back into the turn. The precise sequential location of IDK in turn-beginnings as “sequence-structurally important places in conversation” (Schegloff, 1987, p. 71) is decisive for its working as a preface through which respondents alert co-participants to the dispreferred nature of their incipient responsive action. The morphophonologic reduction and semantic bleaching of IDK indicate that it is being used as a routinized (or: grammaticized, cf. Hopper and Traugott, 2003) particle-like element rather than a subject-verb-negation combination. We will return to this in the discussion (see section “Discussion and conclusion”).

Respondents’ verbal and gaze conduct are assembled in time in a way that gaze aversion either starts in the transition space, i.e., prior to the delivery of IDK, or simultaneously with the onset of IDK, but not later than that. More precisely: When there is no gap between the sequence initiating action and the response, gaze aversion coincides with the IDK preface of the response (ex. 3), i.e., with the verbal start of the responsive turn; by contrast, when gaps or other elements such as ‘well’-prefacing further delay the production of IDK and the response, then gaze aversion tends to start prior to IDK (ex. 4, 5, 6, 7), that is: It tends to start shortly after the end of the question turn (for an exception see ex. 10 below). This observation, though in need of fine-grained analysis based on a larger amount of cases, is roughly in line with Kendrick and Holler’s (2017) finding that gaze aversion begins most frequently 100 ms after the first possible completion point of the question (even in the case where questioning turns reach multiple possible completion points). In all of the examined cases, gaze then remains averted during IDK and into the responsive turn, and typically returns to the prior speaker toward the end of the response. As we will see in what follows, the observed gaze trajectory and its temporal relation to verbal conduct is recurrent across the languages and action contexts studied. Though there are exceptions to this, it is a strong tendency observed in the data.

### Prefacing Dispreferred Responses to Proposals, Assessments, and Informings

While we have so far focused on the question-answer adjacency pair, particle-like uses of IDK combined with gaze aversion are also found in other contexts of incipient dispreferred actions. In this section, we show that the practice of prefacing a dispreferred response with [IDK + gaze aversion] is generalizable across a range of sequence-types, being recurrently found in our data in responses to proposals, assessments, and informing. For reasons of space, we here limit ourselves to illustrating each of these action sequences by one or two examples taken from the languages studied.

#### Proposals

Let us start with two illustrations of IDK in responses to proposals. Couper-Kuhlen (2014) suggests that proposals can be distinguished from similar types of actions (such as suggestions, requests, and offers) in that they are used when the activity is framed as benefiting both speaker and hearer. Excerpt (8) shows an example from French. Daniela and Penny are talking about a joint assignment they have to do for one of their university professors. Daniela proposes that they should go and make an appointment with the professor to discuss some details of the assignment (l.01–02). Daniela gazes at Penny throughout the excerpt.

**Figure:**
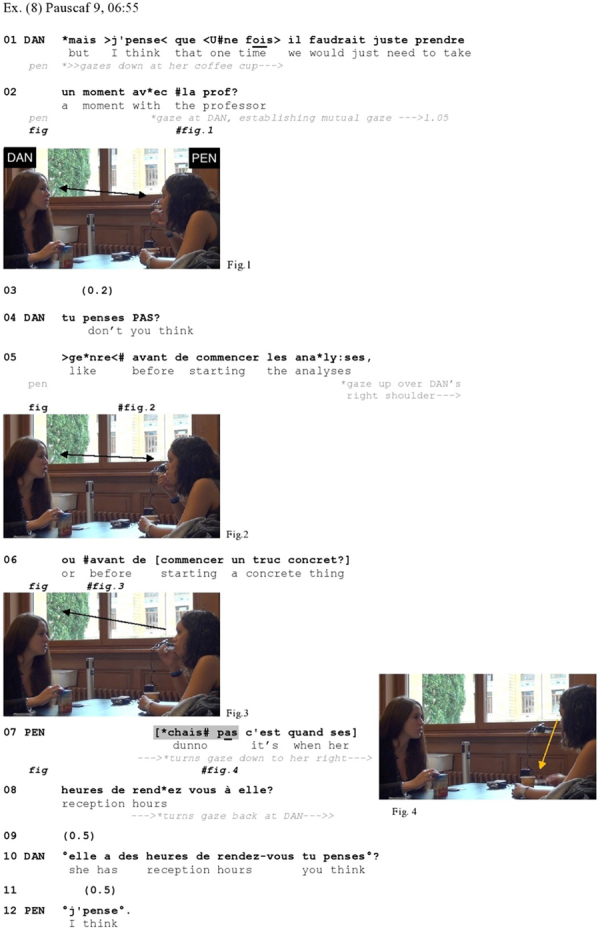


In the face of Penny’s lack of response to her proposal (l.03), Daniela increments it (l.04–06) in pursuit of a response, providing further details about when, in the course of their work-process, they should go to see the professor. It is only here that Penny reacts, yet she does so with a disaligning action: instead of responding to the proposal, she asks if the professor has reception hours (l.07), which ensues in a complex side-sequence (l.07 and following) extending beyond the cited excerpt. Her *chais pas* here introduces an action that is structurally disaligned with the preceding proposal; therefore it can be seen as signaling a sequential disjuncture. Note that Penny’s response is not preceded by any hesitation markers or silence or turn-initial particle other than the IDK, but still comes in a much delayed way in regard to the recognition point of the Penny’s sequence initiating action, the first formulation of which was offered in lines 01–02 and then re-cast in line 04. While Penny had turned her gaze on Daniela in the course of the latter’s proposal (Figure 1) and had maintained it on her throughout part of the re-launch (Figure 2), she then first turns her gaze up over Daniela’s right shoulder right before Daniela’s turn extension reaches a transition relevance place (l.05), and subsequently turns it down toward her right simultaneously with her production of *chais pas* (Figure 4, l.07). Her gaze aversion starts here quite in advance of her response (l.05), and the relatively late delivery of that response in relation of this gaze conduct may be due to Penny’s momentary inability to respond verbally, as she is licking off her coffee spoon (see Figure 2) and takes it out of her mouth only immediately before the delivery of *chais pas* (Figure 3). In other words, gaze aversion is here deployed in a much premonitory manner to the later verbal projection of a dispreferred response through IDK.

So far, we have discussed excerpts in which the recipient actively averts her gaze away from the prior speaker before or concurrently with the IDK token. There are also instances in our data where the respondent’s gaze is already averted from the prior speaker during the speaker’s sequence-initial action, especially when participants are engaged in other activities; in these cases, the respondent’s gaze simply remains averted with the delivery of IDK. Excerpt (9) shows such a case from the Mandarin data where Qun and Str are playing a puzzle (see Figure 1) and are making plans about how to go to a recording appointment together afterward (lines 01–08). The sequence starts with Qun checking whether she understood their plan correctly, namely that they are ‘going there together’ (l.01).

**Figure:**
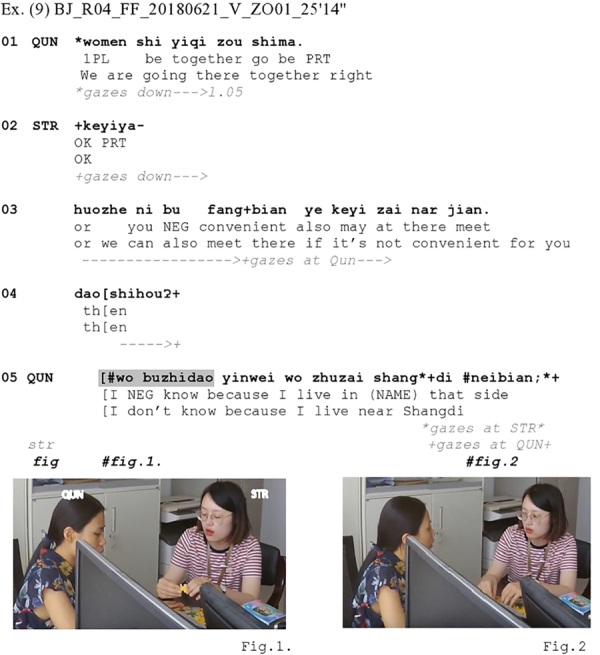


After Qun requests confirmation of her understanding that they are going to the recording studio together (l.01), Str first offers such confirmation (l.02) but then proposes an alternative arrangement which is that they could go separately and directly meet there (l.03). Overlapping with Str’s turn extension (l.04), Qun produces *wo bu zhidao* ‘I don’t know’ (l.05) prefacing an account of her rejection to Str’s proposal (line 06; the ‘wo’ [uo] is here reduced from a diphthong to a schwa). Here, the account in itself does the rejection: When mentioning that her place (the neighborhood named Shangdi, l.06) is far away from the recording studio, Qun implies that going there separately would incur more transportation costs than sharing a taxi. It is noteworthy that, when Qun seeks Str’s confirmation and when Str makes the alternative proposal, Qun looks down at the puzzle (l.01–04). She keeps her gaze down away from Str when she produces *wo bu zhidao* ‘I don’t know’ in line 5 (Figure 1) but then briefly looks at Str toward the end of her account (Figure 2). Here, then, the respondent’s gaze is not actively averted, but merely *remains* averted from the recipient, throughout most of the dispreferred response, yet just as in the prior excerpts returns to the recipient toward the end of that response. So, again, the excerpt shows the same response-initial configuration involving IDK plus gaze averted from recipient as a preface to a dispreferred response. Here, it prefaces the rejection of a proposal.

#### Assessments

Turning now to disagreeing responses with assessments, consider Excerpt (10) from the Czech data. This is taken from a TV talk show in which the host (E. Kočičková, in the middle of Figure 1) leads a talk on homosexuality. The excerpt comes from the beginning of the show, after the host had mentioned that she would have liked to have both genders represented among her guests, but that her ‘female adventures’ did not have the courage to show up (l.01–03). This is produced as an informing containing a negative assessment, to which the male guest Špaček reacts (l.05–06) with a *já nevím* prefaced response.

**Figure:**
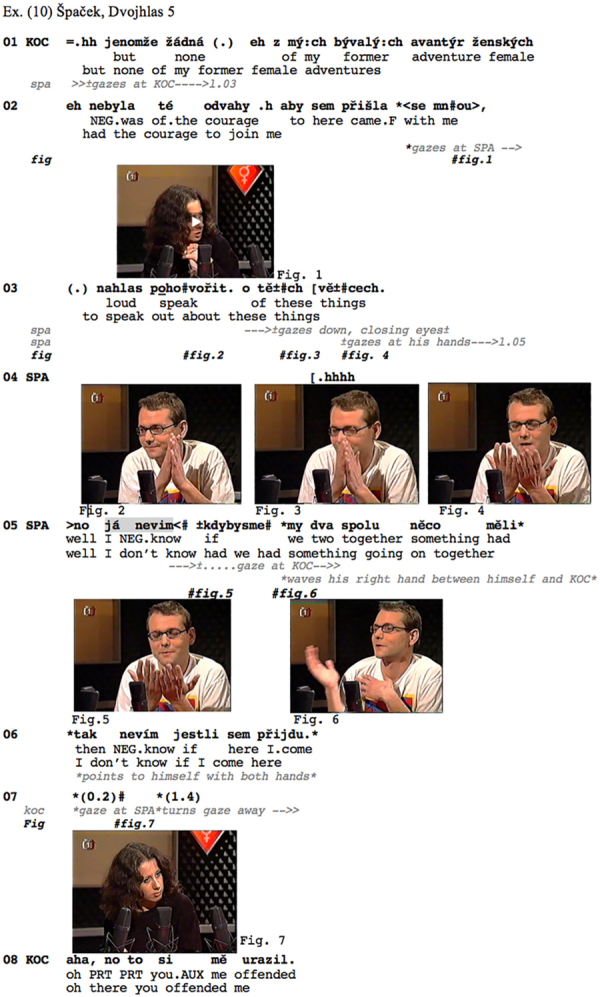


Clearly, the host’s display of disappointment (l.01–02) about not having found a female to sit in the show projects recipients’ affiliation (a shared stance) as a relevant next. Furthermore, it contains a negative assessment. Her *žádná z my:ch bývalý:ch avantýr ženských nebyla té odvahy.h aby*… (l.01–02) – literally ‘none of my former female adventures was of the courage to…’ – involves both an informing and an assessment: While she informs about her former adventures not having come to the show, she also qualifies their not coming as a lack of courage: The assessing element in her turn can be heard as synonymous with the canonical assessment ‘they were too coward to…’. This is what Špaček orients to in his response, in line 05. Rather than responding to the informing, for instance by means of a display of change of knowledge (Heritage, 1984) or by ‘acknowledging’ the informing (Thompson et al., 2015), Špaček displays strong disaffiliation with the host’s stance as well as disalignment with her negative assessment: He counters that assessment by displaying understanding of the women that purportedly refused to participate in the show: Had he been in the same situation as these women, i.e., having had a liaison with the host, he would not have participated either. He thereby disqualifies the idea that these women lacked courage, i.e., were cowards. The *já nevím* ‘I don’t know’ hence occurs as a preface to both a disaffiliating (in terms of stance) and a disaligning (in terms of the structural organization of actions) response, of which the dispreferred nature is further highlighted by the turn-initial *no*, roughly corresponding to English ‘well’ (Müllerová, 1996; see footnote 11 above; Auer and Maschler, 2016), itself preceded by a lengthy and heavy inbreath on the part of the recipient. Just like in Excerpt (6) above, the speaker’s use of the first person pronoun *já* ‘I’ – usually dropped in spoken Czech – works here as a further delay component to the dispreferred response. Furthermore, the whole stretch *no já nevím* is produced with notable speed-up of tempo, being prosodically downgraded, which further adds to its being heard as a preface^12^. The response is then treated as disaligning, and even disaffiliative by the host (l.08), who overtly reproaches to Špaček to have offended her.

The *já nevím* is part of a particularly prominent verbal-bodily assembly here, involving not only the respondent’s gaze but also his hands. During her informing, the host’s gaze wanders between her two guests and at the table in front of her, but toward the end of her turn, she turns her gaze to Špaček, apparently recruiting him as the next speaker (cf. Lerner, 2003; see already Kendon, 1967), which ensues in the establishment of mutual gaze (Figure 2, l.10). In an anticipatory manner, Špaček starts turning his gaze away before the end of Kočičková’s turn (Figures 3, 4, l.03/4), but after the gist of her turn (the negative assessment) has become recognizable (see Broth and Keevallik, 2014; Pekarek Doehler, 2021b, forthc, for how the recognition point of a turn/action in progress may affect the timing of responsive actions, both verbal and embodied). Toward the end of the host’s turn, he shortly closes his eyes (Figure 3, l.03), pulls his hands toward his face, opens his palms and starts gazing at them (Figures 4, 5, l.04/5). His gaze aversion hence precedes the delivery of the *já nevím*, is maintained during that delivery and further into the turn and returns to Kočičková only in the further course of that turn (Figure 6, l.05). This gaze trajectory converges with the evidence provided in the prior excerpts (see in particular section “Intermediate Summary”). In ways similar to what we have observed in dispreferred responses to questions (see section “Prefacing Dispreferred Responses to Questions” above), distinctly strong inbreath, gaze aversion, gesture, *no* ‘well’ and *já nevím ‘*I don’t know’ work in a minutely, step by step assembled way, to incrementally build up the incipient dispreferred response.

#### Informings

Further contexts where we find the focal multimodal assembly is in responses to informing, especially those that confer the speaker’s stance toward the reported state of affairs. Following Thompson et al. (2015, p. 51), we “use the term ‘informing’ to designate the action done when a speaker’s turn is constructed to provide information to a non-knowing recipient such that they become (more) knowing.” ‘Informing’ is hence a cover-term that may include actions such as announcements or news deliveries. As the authors show, respondents generally react to informing by indexing a shift in their epistemic stance, from not or less knowing to more knowing, for instance by means of the delivery of a newsmark (Heritage, 1984, pp. 339–349).

The Romanian Excerpt (11) provides a first illustration. Ana, who is world amateur champion of paragliding and skydiving, just informed Greta that a skydiving contest would take place in August in the city of Dara, where both are working. Ana’s informing then continues with her naming the competitions that she would take part in later, in September (l.01 ff.):

**Figure:**
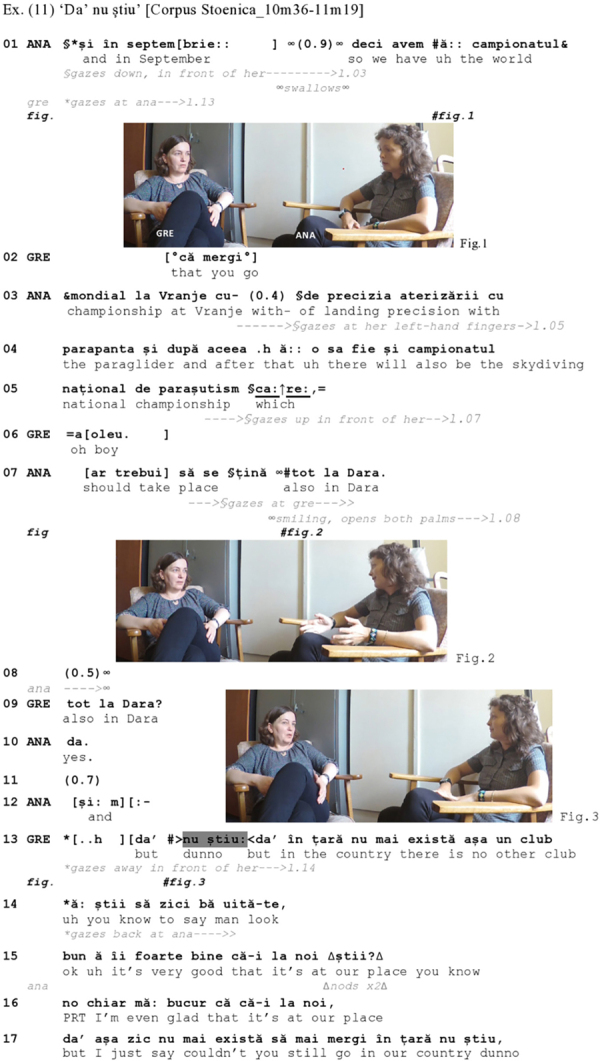


From line 01 to 07, Ana informs Greta about two competitions that she is planning to do in September. Most of the informing is produced in a rather non-committal way, Ana gazing either down (Figure 1) or at her left-hand fingers, while enumerating the respective contests. Her informing though becomes livelier when she announces that the skydiving national championship should take place also in Dara (l.07), just as the other contest planned for August. Here, Ana switches her gaze to Greta, smiling at her, displaying satisfaction with the latter piece of news (Figure 2). Vocally, also, the delivery of this piece has been made in a dramatic fashion, as Ana changes her voice quality and produces the relative pronoun *care* ‘which’ (l.05) with a prosodic emphasis, marked high rise in pitch and sound stretches, projecting thereby a noteworthy information to come up next. In short, Ana displays a clearly positive, if not enthusiastic stance with regard to the news she is delivering (on news delivery sequences, see Maynard, 1997).

Greta, however, does not affiliate with Ana’s stance, and the pause at line 08 projects a potential dispreferred reaction on her part (note the contrast to her response in line 06). Through her phrasal repeat of *tot la Dara* ‘also in Dara’ (l.09) with rising intonation, she first initiates repair on Ana’s turn by asking for a confirmation of the place where the championship should be held, and, after receiving such confirmation (l.10), keeps silent again (l.11), displaying no verbal, prosodic or embodied affiliation with her interlocutor’s stance.

Greta’s dispreferred reaction becomes clear after this short side sequence as she takes up the floor again and produces an extended turn [l.13–18 – an unrelated clausal response (see Thompson et al., 2015, p. 61)], asking Ana if there are not any other places in the country, apart from Dara, where this championship could be held. Her response is dispreferred not in the sense that it does not treat the information provided as news, but in that it denies the positive valence of the news presented. The dispreferred nature of her turn is foreshadowed by the particle *da’* ‘but,’ morphophonologically reduced (from *dar* to *da’*), itself preceded by a marked in-breath (l.13). Together with IDK (*nu ?tiu*), which is delivered with speed-up tempo and prosodically latched to what follows, these push the response proper further back into the turn. Also, Greta averts her gaze from Ana exactly with the strong inbreath and keeps it averted until line 14, while she was before constantly looking at Ana (l.01–12). Thompson et al. (2015, p. 14) note: “There exists […] a strong norm for at least acknowledging, or ‘receipting,’ an informing; in this sense, a ‘dispreferred’ response to an informing would be to not acknowledge the information as an informing at all.” This is exactly what Greta does: Rather than acknowledging the informing, Greta goes on questioning the relevance of holding the championship at Dara. Finally, note that Greta herself orients to the dispreferred nature of her own turn as she provides two parenthetical comments on the fact that she appreciates nevertheless that the championship is organized in Dara (l.15–16), thereby somewhat mitigating her rather strong disaffiliative reaction to her coparticipant’s stance.

Excerpt (12) illustrates a disagreement with what can be qualified as an informing that is strongly asserted, i.e., offered as a claim about a state of affairs; as such, it resembles what Vatanen et al. (2020, p. 6) qualify as an ‘assertion’. It occurs in a context of prolonged disagreement between the coparticipants, where it conveys the speaker’s stance toward the talked-about issue. Luo and Yan are commenting on the performance of a well-known Chinese actress Zhang Yizi (referred to as ‘she’ in line 01) in a movie they have recently watched together.

**Figure:**
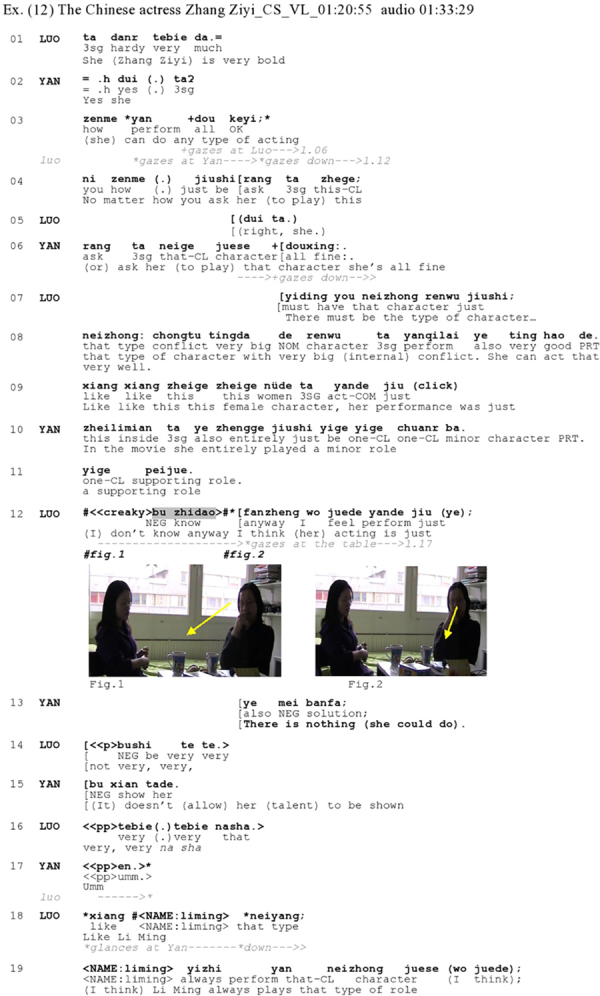


In lines 01–06, Luo and Yan affiliate with each other in producing positive assessments of the boldness and versatility of the actress Zhang Ziyi. But in lines 07–12, Luo pivots his evaluative stance. He produces a qualified assessment that the actress plays particular types of characters with large internal conflicts well (l.07–08), thereby somehow disaligning with Yan’s prior assertion that ‘(she) can do any type of acting’ (l.03). In line 9, Luo makes a syntactically incomplete ‘off-record’ critical assessment of the performance of Zhang Ziyi in the specific movie that Luo and Yan recently watched. Syntactically incomplete assessments in Mandarin conversation have been documented to perform ‘off-record’ negative assessments (Li, 2016; see also Park and Kline, 2020 for a similar use in English), which can here possibly also be observed from Luo’s gaze aversion from Yan. Then, in lines 10–11, Yan asserts that the actress plays an ‘entirely’ minor role in the movie, possibly in an attempt to discard the idea that one could solidly judge the actress’ talents based on that one movie. The extreme-case formulation *zhengge* ‘entirely’ (Pomerantz, 1986; Whitehead, 2015) expresses Yan’s “unstated disagreement” with Luo (Pomerantz, 1984, p. 76). Immediately after Yan’s turn, Luo produces *bu zhidao* ‘(I) don’t know’ (l.12) followed by another negative assessment of the actress (ll. 12, 14, and 16). It is interesting that Luo averts her gaze from Yan already from line 03 on, possibly in a premonitory way to her subsequent verbally expressed disagreeing stance, which she builds up incrementally: During Yan’s turn in line 03, Luo shifts his gaze from Yan down at the mug on the table and keeps his gaze down during almost the entire sequence (Figure 1), lowering it further immediately after *bu zhidao* ‘(I) don’t know’ (Figure 2, l.12). So, here, Luo also seems to deploy *bu zhidao* ‘(I) don’t know’ (l.12) and a lack of mutual visual engagement with Yan (Figure 4) to project his disagreement with Yan’s immediately preceding assertion and the evaluative stance expressed therein (ll. 10–11).

In this section, we expanded our prior observations documenting that the focal assembly of gaze aversion plus IDK in turn-initial position represents a practice that can be found not only with the prototypical adjacency pair of the type question-answer, but also in a range of other action contexts, such as responses to proposals, assessments and informing – specifically informing that convey the speaker’s stance. The practice is hence deployed in locally functional ways across a range of sequence types. The excerpts cited have also shed further light on the trajectories of recipients’ gaze respective to the prior speaker and their own verbal conduct. They confirmed the consistent (re)turning of recipients gaze to the prior speaker toward the end of the responsive turn – even when participants are involved in multiactivity, such as playing a puzzle (ex. 10). The excerpts also showed that recipients’ gaze aversion from prior speakers typically occurs in the transition space or simultaneously with the response onset; only rarely does it occur during the preceding speaker’s turn, but in any case after the recognition point of the prior action, and hence of the conditionally relevant next action. The onset of such gaze conduct has practical interactional import: For instance, when it precedes the end of the sequence initial turn (ex. 10), it may be a way for respondents to project a dispreferred response in a premonitory way while circumventing overlap; when it occurs in the transition space while the recipient is unable to speak (e.g., while eating, ex. 8), it may be a way to warrant early projection of aspects of the incipient responsive action. In this sense, respondents can be seen to minutely assemble, on-line, their gaze conduct and their vocal conduct in locally functional ways for all practical purposes.

## Discussion and Conclusion

In this paper we examined how an assembly of verbal and embodied conduct is related to one of the basic organizational principles of social interaction: preference organization. We documented a recurrent bimodal practice in which speakers deploy IDK in combination with gaze aversion in turn-initial position to project an incipient dispreferred response: Through this practice, speakers retroactively display resistance to the constraints set by and/or disagreement with the stance conveyed by the immediately preceding action, and prospectively project a dispreferred response. We showed that this practice occurs across a diverse set of languages and a variety of sequence types. The findings call for further detailing based on a more extensive collections for some of the languages (namely: Romanian), and comparison across a larger set of languages, and specifically of culturally more diverse participant groups: Despite the diverse language (sub-)families represented in this study, all of our data stem from post-industrial societies. With these limitations in mind, we spell out, in the following paragraphs, some implications of our findings.

We started out by choosing to work on a specific negative epistemic expression involving 1st person and the negated verb of knowing. It turned out that in the focal sequential position – turn-initial position in responses to various initiating actions –, the structure could be variably prefaced with additional materials, such as clicks, hesitation tokens, and particles such as *ben* in French or *no* in Czech. Importantly, it was often morphophonologically reduced and sometimes prosodically downgraded (by lower volume or speed up of tempo) in all of our languages. These features suggest a particle-like working of the IDK construction, which appears to have routinized in all of the studied languages into an interaction-organizational device.

This finding adds to existing research on IDK, evidencing how prefacing uses, far from being limited to functioning as epistemic hedges (e.g., Weatherall, 2011), are implicated in the management of the multimodal infrastructure of social interaction, and specifically in the prefacing of dispreferred responses. Heritage and Sorjonen (2018) show that turn-initial objects in first position manage the connection of the current turn to its immediately preceding one, and those in responsive position may be used for resisting the constraints set by the first turn on the second position speaker. This study develops this line of research in two ways. First, we showed that in addition to particles such as *well, ben, nu/no* documented in previous research, phrases such as ‘I don’t know’, concurrent with gaze aversion, are a common occupant in turn-initial position across five distinct languages. This adds to our knowledge of the *type* of turn-initial objects, and particularly suggests that these may include not only linguistic but also bodily visual aspects as part of methodic turn-construction practices. Second, by showing that and how the multimodal practice consisting of IDK and gaze aversion is deployed to preface an incipient dispreferred response in a range of sequence types, this study demonstrates that a practice like this can be applicable across a variety of responsive actions, possibly irrespective of any precise type of the initiating action.

The findings further add to existing knowledge on gaze in interaction, expanding specifically on the results offered by Kendrick and Holler (2017), and more recently Robinson (2020), who demonstrate that dispreferred answers to polar questions tend to correlate with respondent’s gaze averted from the questioner. Clearly, gaze aversion also in animals is related to submission and avoiding confrontation, so this gaze aversion in dispreferred sequences is valid across species and has its natural origin in non-confrontational behavior in general (see Kendrick and Holler, 2017). The findings presented here amplify prior observations that gaze-aversion is found with dispreferred responses by showing that this association is valid across a range of sequence and action types, extending to responses beyond those provided to polar questions, and that this is the case across genetically and typologically different languages. Based on these findings, we suggest that by paying close analytic attention to the multimodal make-up of turn formats we might arrive at a more fine-grained understanding of the methodic multimodal practices involved in turn construction and action formation, such as the temporal unfolding of gaze behavior *in relation* to verbal and vocal conduct, and the uttering of specific turn-initial verbal phrases *in concert* with precise embodied conduct.

Finally, the type of multimodal sequential analysis we conducted here allowed us to evidence gaze *trajectories* associated to dispreference. The data show that gaze aversion most typically starts after the end of the sequence initial action – a result that converges with Holler and Kendrick’s (2017) earlier finding for responses to polar questions. Additionally, the present study evidences that such aversion starts either in the transition space if there is a gap between turns, or else concurrent with response onset; it also shows that respondents’ gaze tends to revert to the prior speaker toward the end of the responsive turn, and that these gaze trajectories hold across different sequence types and languages. The fact that gaze aversion is rarely found to overlap with the end of the sequence initial turn is intriguing in light of prior research showing that response planning begins as early as possible, and sometimes even during the turn-in-progress (e.g., Levinson and Torreira, 2015) – which might ensue in the production of responsive turns in overlap with sequence initiating turns (Pekarek Doehler, 2021b, forthc) or responsive embodied action (such as affirmative nods, De Stefani, 2021) before the end of initiating turns and actions. Ultimately, by evidencing how grammar and body interface in related ways across languages, the findings open a window onto cross-linguistic, cross-modal, and cross-cultural regularities in human interactional conduct.

## Transcription Conventions for Embodied Conduct

**/±± Symbols such as these indicate start and end of embodied conduct
*—–> l.12 Continuation of the described embodied conduct until line 12 of transcript.
——>* End of the described embodied conduct
*——> > Continuation of the described embodied conduct until end of excerpt
# Indicates the location of a figure in the verbal transcript

## Data Availability Statement

The original contributions presented in the study are included in the article/supplementary material, further inquiries can be directed to the corresponding author/s.

## Ethics Statement

Ethical review and approval was not required for the study on human participants in accordance with the local legislation and institutional requirements. Czech data comes from TV talk shows but converges with the data in the other languages in that it consists of unscripted interactions that unfold spontaneously. As the latter data comes from the public domain, it neither needs anonymization nor consent for publication. Names in all other data have been anonymized, and written informed consent has been obtained from all participants for use and publication of the materials, including the video frames.

## Author Contributions

SPD has done the main research work on this manuscript, in collaboration with HP-Y. All co-authors contibuted with data analysis and detailed discussions of the various versions of the manuscript. All authors contributed to the article and approved the submitted version.

## Conflict of Interest

The authors declare that the research was conducted in the absence of any commercial or financial relationships that could be construed as a potential conflict of interest.

## Publisher’s Note

All claims expressed in this article are solely those of the authors and do not necessarily represent those of their affiliated organizations, or those of the publisher, the editors and the reviewers. Any product that may be evaluated in this article, or claim that may be made by its manufacturer, is not guaranteed or endorsed by the publisher.

## Footnotes

1 Some of the studied languages have so far remained largely unexplored in an interactional linguistics approach (Romanina; Czech; but see Oloff and Havlík, 2018).

2 Stivers (2010, p. 2778) writes: “non-answer responses included not only ‘I don’t know’ and ‘I can’t remember,’ but also less committal ‘Maybe’ or ‘Probably’ responses and less direct answers”.

3 Other factors may play into preference organization (Pomerantz and Heritage, 2013), such as the sub-type of sequence-initial action (e.g., for certain offers, accepting might be a dispreferred action; Schegloff, 2007, p. 60) or the speaker’s entitlement to perform that action (Curl and Drew, 2008).

4 Following Stivers et al. (2011) we use alignment to refer to the “structural level of cooperation” (2011, p. 20) and see aligning responses as ones that “cooperate by facilitating the proposed activity or sequence; accepting the presuppositions and terms of the proposed action or activity; and matching the formal design preference of the turn” (2011, p. 21). Affiliation in turn relates to the “the affective level of cooperation,” in that “affiliative responses are maximally pro-social when they match the prior speaker’s evaluative stance, display empathy and/or cooperate with the preference of the prior action.” (Stivers et al., 2011, p. 21).

5 The Czech data come from Korpus DIALOG 2.0 (2020). Online: <http://ujc.dialogy.cz>. The corpus, consisting of 200 recordings (and related transcriptions), contains a total of 1,188,513 words. There is a total of 856 speakers, 305 of which uttered more than 1000 words.

6 Czech, Mandarin, and Romanian are pro-drop languages with possible null subjects (e.g., Huang, 1989, for Mandarin). That is, the subject pronoun ‘I’ may be dropped, creating reduced form such as *bu zhidao* ‘don’t know.’ The French *je ne sais pas* has a discontinuous NEG morpheme (*ne…pas*). In spontaneous spoken language use, the *ne* is commonly dropped, and the expression can further be reduce based on amalgamation of *je* and *sais*. In Hebrew the present tense does not mark person, but only gender and number.

7 Note, that contrary to the typical shrug described by Streeck (2009, p. 189sq), Ana here deploys what Kendon (2004, p. 251) refers to as ‘vertical palm’ « used in contexts where the speaker indicates an intention to halt the current line of action ». This gesture may here be seen as conferring Ana’s disengagement from the ongoing line of action and topic, and not only from her response.

8 On multiple questions see Clayman and Heritage, 2002; Linell et al., 2003; Pekarek Doehler, 2021a, forthc.

9 Furthermore, it formally displays a preference for a type-conforming response, i.e., a response of the yes-no type. Yet, given its situational context, the question works as an invitation to elaborate rather than ask for a simple ‘yes’ or ‘no’ answer.

10 According to Müllerová, 1996, p. 226: “In response to a question or to a preceding turn in general the speaker signals [i.e., projects] by the word *no* (…) his or her doubt, reservation, resignation; negative statement, rejection.” (our translation).

11 Figures 1 and 2, in the transcript, show shows each participant in a different camera capture but from the preceding and subsequent interaction it becomes clear that these captures present the participants’ gazing at each other.

12 It is important to note that the subsequent if-clause, in line 5, cannot grammatically stand as the complement clause to IDK: in Czech, *jestli* (*if* or *whether*) is a typical conjunction for complement clause following *(já) nevím*; the conditional conjunction *kdyby* used here, by contrast, does not work to introduce a complement clause after *(já) nevím*; here, it marks the start of a stretch of talk that is syntactically independent of the preceding *já nevím*.
